# A Comprehensive Nomogram Integrating Phonocardiogram and Echocardiogram Features for the Diagnosis of Heart Failure With Preserved Ejection Fraction

**DOI:** 10.1002/clc.70022

**Published:** 2024-10-28

**Authors:** Linchun Cao, Xingming Guo, Kangla Liao, Jian Qin, Yineng Zheng

**Affiliations:** ^1^ Department of Radiology The First Affiliated Hospital of Chongqing Medical University Chongqing PR China; ^2^ Department of Cardiology The First Affiliated Hospital of Chongqing Medical University Chongqing PR China; ^3^ Department of Cardiology People's Hospital of Fengjie County Chongqing PR China; ^4^ Key Laboratory of Biorheology Science and Technology, Ministry of Education, College of Bioengineering Chongqing University Chongqing PR China; ^5^ State Key Laboratory of Ultrasound in Medicine and Engineering Chongqing Medical University Chongqing China; ^6^ Medical Data Science Academy Chongqing Medical University Chongqing China

**Keywords:** echocardiography, heart sound, HFpEF, nomogram, predictive model

## Abstract

**Background:**

Heart failure with preserved ejection fraction (HFpEF) is associated with high hospitalization and mortality rates, representing a significant healthcare burden. This study aims to utilize various information including echocardiogram and phonocardiogram to construct and validate a nomogram, assisting in clinical decision‐making.

**Methods:**

This study analyzed 204 patients (68 HFpEF and 136 non‐HFpEF) from the First Affiliated Hospital of Chongqing Medical University. A total of 49 features were integrated and used, including phonocardiogram, echocardiogram features, and clinical parameters. The least absolute shrinkage and selection operator (LASSO) regression was used to select the optimal matching factors, and a stepwise logistic regression was employed to determine independent risk factors and develop a nomogram. Model performance was evaluated by the area under receiver operating characteristic (ROC) curve (AUC), calibration curve, decision curve analysis (DCA), and clinical impact curve (CIC).

**Results:**

The nomogram was constructed using five significant indicators, including NT‐proBNP (OR = 4.689, *p* = 0.015), *E*/*e*′ (OR = 1.219, *p* = 0.032), LAVI (OR = 1.088, *p* < 0.01), D/S (OR = 0.014, *p* < 0.01), and QM1 (OR = 1.058, *p* < 0.01), and showed a better AUC of 0.945 (95% CI = 0.908–0.982) in the training set and 0.933 (95% CI = 0.873–0.992) in the testing set compared to conventional nomogram without phonocardiogram features. The calibration curve and Hosmer–Lemeshow test demonstrated no statistical significance in the training and testing sets (*p* = 0.814 and *p* = 0.736), indicating the nomogram was well‐calibrated. The DCA and CIC results confirmed favorable clinical usefulness.

**Conclusion:**

The nomogram, integrating phonocardiogram and echocardiogram features, enhances HFpEF diagnostic efficiency, offering a valuable tool for clinical decision‐making.

## Graphical abstract

A total of 204 patients and 49 features were collected. After splitting the population into training and test sets according to a ratio of 7:3, the LASSO and logistic regression was used to select the final five factors for a nomogram. The AUC for the nomogram model is 0.945, while the AUC for the echocardiographic parameters combined with NT‐proBNP in the diagnostic model (*E*/*e*′ + LAVI + NT‐proBNP) is 0.887. In conclusion, combining phonocardiogram, echocardiogram features, and clinical parameters can effectively improve the diagnostic efficiency of HFpEF.

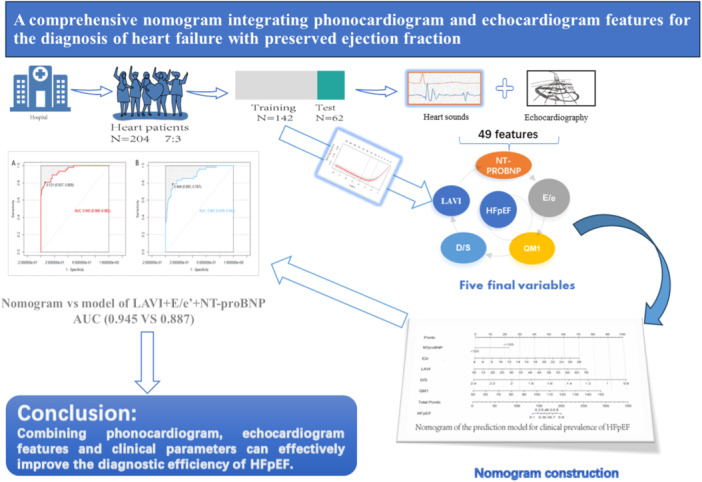

## 1 Introduction

Heart failure (HF) is a progressive, multifactorial, and heterogeneous clinical syndrome characterized by symptoms and signs that result from any structural or functional impairment of ventricular filling or ejection of blood. It is categorized based on ejection fraction (EF) into HF with reduced EF (HFrEF, left ventricular ejection fraction [LVEF] ≤ 40%), HF with mildly reduced EF (HFmrEF, LVEF 41%–49%), HF with improved EF (previous LVEF ≤ 40% and a follow‐up measurement of LVEF > 40%), and HF with preserved EF (HFpEF, LVEF ≥ 50%) [1, 2, 3, 4], HFpEF can be further classified into HF with normal EF (HFnEF, 50% ≤ LVEF ≤ 65%) and HF with supra‐normal EF (HFsnEF, LVEF > 65%) [5, 6]. Among them, HFpEF constitutes 50% of HF cases [7], which is correlative with ageing, hypertension, obesity, and characterized by an elevated left ventricular filling pressure [8], with comparable rates of readmission and mortality to HFrEF [9]. Due to the different treatment approaches for HFpEF compared to HFrEF, and the irreversible nature of HF progression, early and accurate diagnosis of HFpEF is particularly crucial for slowing the progression of the condition. However, the pathogenesis of HFpEF is intricate, involving various physiological pathways including systemic inflammation [10, 11], inadequate release of natriuretic peptides, activation of the renin–angiotensin–aldosterone system (RAAS), and sympathetic nervous system [12]. Clinical diagnosis has always been challenging due to the high heterogeneity of HFpEF and lack of specific early symptoms and signs [13, 14]. Invasive hemodynamic measurements serve as the gold standard for diagnosing HFpEF, defined by a pulmonary capillary wedge pressure (PCWP) ≥ 15 mmHg at rest or a left ventricular end‐diastolic pressure (LVEDP) ≥ 16 mmHg, and a PCWP ≥ 25 mmHg under stress [15, 16]. However, the invasive nature of the gold standard limits its clinical application. Echocardiography can provide valuable insight into the pathophysiology and underlying phenotypes of HFpEF, facilitating its diagnosis. Echocardiographic parameters, when combined with serological biomarkers like N‐terminal pro‐brain natriuretic peptide (NT‐proBNP), are recommended in current European HF guidelines as highly sensitive but moderately specific markers of HF [17]. Although there is growing interest in utilizing exercise stress echocardiography for early HFpEF diagnosis, its operational complexity and limited patient acceptance have restricted its widespread application [18]. Currently, the H2FPEF [19] and HFA‐PEFF [20] scoring systems serve as the primary methods for diagnosing HFpEF among physicians, offering advantages such as non‐invasiveness, repeatability, and efficiency. Although studies have shown that both the HFA‐PEFF and H2FPEF scoring systems have strong diagnostic efficacy for HFpEF, a single system still has clinical gray areas [21, 22]. Using both systems together can improve diagnostic performance, but it is more complex and cumbersome, increasing the medical burden. Therefore, there is a need for a simpler and highly effective noninvasive diagnostic tool for HFpEF that remains clinically significant.

The basic diagnosis of HFpEF can be based on phenomena such as hemodynamic‐related congestion, myocardial stiffness, or elevated filling pressures, and the changes in the features of phonocardiogram (PCG) are a direct reflection of alterations in cardiac hemodynamics. Therefore, phonocardiography can overcome the limitations of human auditory perception in cardiac auscultation for HF screening, facilitating the identification of irregular patterns through visual examination and the extraction of objective features like amplitudes, frequencies, and time intervals in the heartbeat. Zheng et al. [23] have shown the potential of PCG feature‐based least square support vector machine (LS‐SVM) for differentiating the patients with chronic HF from the healthy. Liu et al. [24] He proposed an automated HFpEF diagnostic method using extreme learning machine (ELM) classifiers, employing the ratio of diastolic to systolic duration and multifractal features from PCG signals. The method demonstrated the effectiveness of heart sounds in HFpEF diagnosis. Luo et al. [25] conducted an initial study to examine the correlation between PCG features derived from acoustic cardiography and early mitral inflow velocity and early diastolic mitral annular velocity (*E/e*′) obtained from echocardiography in patients with suspected HFpEF. The study showed that the PCG feature, the interval from QRS onset to the first heart sound (QS1), can categorize *E*/*e*′ in symptomatic patients suspected of HFpEF with a diagnostic performance comparable to that of NT‐proBNP. Considering that PCG is also advantageous due to its ease of collection, non‐invasiveness, low cost, and potential for remote monitoring [26], it is expected to provide supplementary information for the auxiliary diagnosis of HFpEF.

Clinical algorithms such as HFA‐PEFF and H2FPEF are currently the most widely accepted and extensively used tools by physicians for diagnosing HFpEF, utilizing not a single parameter but rather a combination of multiple sources of clinical data. However, the diagnostic capacity of clinical algorithms may be limited by discordant or incomplete data, leading to variability and an increased need for invasive confirmatory tests such as right heart catheterization. This adds burden to patients and may result in overlooking individuals who could benefit from treatment. Machine learning or deep learning is well suited to address these technical challenges and has the potential to demonstrate excellent discrimination unreachable with routine clinical data. To enhance the acceptance of the constructed algorithmic model among clinical practitioners, this study proposed to employ a nomogram integrating routinely acquired clinical indicators such as PCG features, echocardiographic parameters, and serum biomarkers to develop a comprehensive and noninvasive diagnostic model for HFpEF. The major advantages of this study are as follows:

1. This study is the first to integrate PCG features and echocardiographic features obtained from routine clinical practices, employing machine learning methods to construct a nomogram for visualizing the diagnosis of HFpEF.
2. A nomogram based clinical scoring system was developed for the diagnosis of HFpEF. This system achieves HFpEF diagnosis by intuitively visualizing various routinely collected clinical indicators while balancing the diagnostic efficacy and interpretability of the model.
3. The nomogram achieved favorable diagnostic performance, emphasizing that the incorporation of PCG features can effectively enhance diagnostic efficacy, validating the effectiveness of heart sound features in diagnosing HFpEF.

## 2 Methods

### 2.1 Study Design and Participants

This single‐center cross‐sectional study was approved by the Medical Ethics Committee of the First Affiliated Hospital of Chongqing Medical University (approval number 2022‐228). It consecutively collected PCG, electrocardiogram (ECG), echocardiogram, and clinical baseline data of inpatients between March 2023 and October 2023, and all study participants provided written consent. The Transparent Reporting of a Multivariable Prediction Model for Individual Prognosis or Diagnosis (TRIPOD) statement [27] was used as a reporting guideline (see Supporting Information S1: Table S1).

Eligible patients were included when: patients aged ≥ 18 years with HF symptoms and/or signs (such as dyspnea, fatigue, fluid retention) and LVEF ≥ 50%. Exclusion criteria comprised high‐degree atrioventricular block, implanted cardiac pacemaker, heart transplantation, severe valvular disease, postoperative artificial valve replacement, congenital heart disease, acute myocardial infarction, cardiomyopathy, constrictive pericarditis, renal failure or dialysis requirement, advanced‐stage active tumors, chronic obstructive pulmonary disease, bronchial asthma, severe anemia (Hb < 60 g/L), poor image quality of echocardiography, and previous LVEF < 50%. An H2FPEF score ≥ 6 or HFA‐PEFF score ≥ 5 was used as a rule‐in criterion for HFpEF [28] (see Supporting Information S1: Methods), and patients with HF‐like symptoms or signs, sensitive to diuretics and vasoactive drugs, and diagnosed by a cardiovascular specialist with 30 years of experience (J.Q.). Patients without HF symptoms or signs but with risk factors (such as hypertension, atrial fibrillation, obesity, diabetes, coronary artery disease, etc.) were included as controls. The study initially enrolled 287 patients. Of these, 34 were excluded due to missing data, 27 did not meet the inclusion criteria, and 22 failed to meet the scoring requirements, resulting in a total of 83 exclusions. Ultimately, 68 HFpEF patients and 136 non‐HFpEF patients with risk factors were included in the study (see Supporting Information S1: Figure S1).

### 2.2 PCG and ECG Feature Extraction

A multi‐channel physiological signal acquisition and processing system (RM‐6240EC, Chengdu Instrument Factory, China) and a heart sound transducer (CXY‐01) were employed to simultaneously capture PCG and single‐lead ECG data for 4–5 min. Participants were instructed to maintain a calm state during the recording, and PCG and ECG signals were sampled at a frequency of 8 and 0.5 kHz, respectively. The collection of PCG and ECG signals was performed by researchers who were blinded to both echocardiography results and the condition of the patients. The raw PCG signals were denoised, including the removal of power frequency noise, bandpass filtering from 10 to 800 Hz, and the application of a previously proposed denoising method to mitigate environmental noise and friction noise from the sensor against the skin [29]. The elimination of high‐frequency noises in the raw ECG is achieved through Savitzky–Golay (SG) filtering, with the polynomial order and window dimension of the SG filter set to 5 and 21, respectively, and the high‐pass Butterworth filter with a third order and a cut‐off frequency of 1 Hz is employed to attenuate any remaining baseline wandering. The down‐sampling and *Z*‐score normalization were applied to both PCG and ECG signals. The hidden semi‐Markov model segmentation algorithm was used to determine the onset points of the first heart sound (S1) and second heart sound (S2), followed by manual adjustments to ensure the accuracy of the results. The extracted features (see Supporting Information S1: Figure S2) are as follows:

1. D/S: the ratio of diastole to systole duration.
2. LVSTc: the ratio of left ventricular systolic time to RR interval. The RR interval indicates the time elapsed between two successive R waves of the QRS signal on the ECG.
3. QS1: time interval from the onset of the Q‐wave on electrocardiogram to the first heart sound.
4. QM1 (EMAT): time interval from the onset of the Q‐wave to the closure of the mitral valve.
5. EMATc: time interval from Q‐wave to the closure of the mitral valve/RR interval.
6. QS2c: time interval from the onset of the Q‐wave to the second heart sound/RR interval.
7. QA2c: time interval from the onset of the Q‐wave to the closure of the aortic valve/RR interval.

### 2.3 Echocardiographic Feature Extraction

Echocardiographic examinations including two‐dimensional measurements, Doppler and tissue Doppler imaging were performed using the GE E95 Echo‐system (GE, Vingmed Ultrasound, Horten, Norway) with the patients in a resting supine position. LVEF and various functional parameters, including the ratio of early mitral inflow velocity to early diastolic mitral annular velocity (*E*/*e*′) (see Supporting Information S1: Figure S3A–C), peak tricuspid regurgitation velocity (TRPV), pulmonary artery systolic pressure (PASP), and global longitudinal strain (GLS) of the left ventricle, along with morphological parameters such as left atrial volume index (LAVI) (see Supporting Information S1: Figure S4A,B), left ventricular mass index (LVMI), relative wall thickness (RWT), and left ventricular wall thickness, were evaluated according to the American Society of Echocardiography and the European Association of Cardiovascular Imaging recommendations during routine clinical care [30]. The average value was obtained from three consecutive cardiac cycles. All stored image data were analyzed by experienced sonographers (K.L. and L.C.), who were blinded to the PCG, ECG data, and patients' conditions.

### 2.4 Clinical Data Collection

The previous diagnoses, examination data, and laboratory tests of all patients during hospitalization were obtained as clinical features from the Electronic Medical Record System of the First Affiliated Hospital of Chongqing Medical University. These features included hospital ID, gender, age, BMI, blood pressure, underlying diseases, smoking history, hemoglobin level, liver and renal function tests, blood potassium level, NT‐proBNP level, glycosylated hemoglobin, thyroid function tests, and types of medications for HF treatment and antihypertensive drugs. Initially, data were checked for integrity and consistency, and any incomplete or inconsistent data were corrected. Then, the study then employed the multiple imputation chain equation method [31], conducting five imputations to address missing values by leveraging all available information. The outcomes from these five imputed datasets were subsequently combined using Rubin's rules. To ensure data validity, a sensitivity analysis was performed using the random forest algorithm, comparing the original data with the imputed data. For practical clinical application, the significant continuous variable NT‐proBNP was dichotomized (NT‐proBNP ≥ 125 pg/mL) based on meaningful criteria derived from the literatures [20, 32].

### 2.5 Feature Selection and Nomogram Development

The features derived from PCG, echocardiogram, and clinical data were combined for dimensionality reduction in the model construction. Firstly, the least absolute shrinkage and selection operator (LASSO) regression was applied to select significant features from the training set, which initially had 49 features, aiming to reduce dimensionality and prevent overfitting. LASSO regression is a data mining method that adds a penalty function to the commonly used multiple linear regression, continuously shrinking the coefficients to achieve the goal of simplifying the model and avoiding multicollinearity and overfitting. Subsequently, multiple forward stepwise logistic regression was then conducted to identify the independent factors that impact the prevalence of HFpEF and construct a nomogram, according to the Akaike Information Criterion. The Box‐Tidwell test was used to evaluate the linear relationship between continuous independent variables and the logit‐transformed values of the dependent variable. The tolerance and variance inflation factor (VIF) were employed to assess multicollinearity.

### 2.6 Statistical Analysis

Continuous variables are reported as mean with standard deviation (SD) or median (25th–75th interquartile range) and count data were expressed as numbers (%). Categorical variables between two groups were compared using the chi‐square test or Fisher's exact test, and continuous variables were assessed using the Mann–Whitney *U* test or independent *t*‐test. The Shapiro–Wilk test was used to determine the normality of the distribution of continuous variables. The receiver operating characteristic (ROC) curves were used to determine the discrimination efficiency of the model, and the DeLong test was used to compare different ROCs. The area under the curve (AUC) and 95% confidence interval (CI) were calculated. The calibration curves and Hosmer–Lemeshow test were employed to assess the goodness of fit of the model. The decision curves analysis (DCA) and clinical impact curves (CICs) were used to measure clinical benefit of the nomogram. Net reclassification improvement (NRI) and integrated discrimination improvement (IDI) were calculated to assess the performance differences between different models. Statistical significance was defined as a two‐tailed *p* value of < 0.05. The nomogram, ROC analyses, DCA, CIC, and RF were performed using “pROC.,” “rms,” “dca.r,” “rmda,” and “randomForest” packages, and all analyses were performed using R software (version 4.3.1) and IBM SPSS 27.

## 3 Results

### 3.1 Study Population Characteristics

The study ultimately included 204 eligible subjects, with 68 individuals diagnosed with HFpEF and 136 non‐HFpEF individuals. The comparison of baseline characteristics between the HFpEF group and the non‐HFpEF group is shown in Table 1. Patients with HFpEF exhibited significantly higher proportions of females, more hypertension, atrial fibrillation, NT‐proBNP elevation, older age, and increased use of antihypertensive medications and diuretics, alongside decreased hemoglobin levels (*p* < 0.05) compared to non‐HFpEF individuals. Transthoracic echocardiography revealed that patients with HFpEF were more likely to have right ventricular dysfunction, higher pulmonary artery pressure, and increased tricuspid regurgitant peak velocity (TRPV). Additionally, they exhibited more pronounced diastolic dysfunction, characterized by higher noninvasive estimates of filling pressure (*E*/*e*′ ratio) and larger left atrial volume index (LAVI) estimates. Additionally, patients with HFpEF had subtle impairment in systolic function as evidenced by lower GLS. The analysis of the PCG features showed that patients with HFpEF had a lower D/S, while LVSTc, QS1, QM1, EMATc, QS2c, and QA2c were significantly increased (Table 1, all *p* < 0.05).

**Table 1:** Baseline characteristics.

Variables	Total (*n* = 204)	Non‐HFpEF(*n* = 136)	HFpEF (*n* = 68)	*p*
Age (years)	65.82 ± 11.28	62.96 ± 11.00	71.54 ± 9.61	< 0.001
Female, *n* (%)	99 (48.53)	59 (43.38)	40 (58.82)	0.038
BMI (kg/m^2^)	24.32 ± 3.14	24.51 ± 2.99	23.95 ± 3.39	0.224
Systolic BP (mmHg)	135.42 ± 18.95	135.29 ± 18.07	135.69 ± 20.74	0.886
Hypertension, *n* (%)	141 (69.12)	84 (61.76)	57 (83.82)	0.001
Hyperlipidemia, *n* (%)	52 (25.49)	39 (28.68)	13 (19.12)	0.140
T2DM, *n* (%)	73 (35.78)	45 (33.09)	28 (41.18)	0.256
AF, *n* (%)	28 (13.73)	5 (3.68)	23 (33.82)	< 0.001
CHD, *n* (%)	101 (49.51)	70 (51.47)	31 (45.59)	0.428
Stent, *n* (%)	41 (20.10)	28 (20.59)	13 (19.12)	0.805
Smoking, *n* (%)	59 (28.92)	45 (33.09)	14 (20.59)	0.063
Hemoglobin（g/L）	133.79 ± 19.87	139.29 ± 16.53	122.79 ± 21.49	< 0.001
BUN (μmol/L)	6.05 (5.10, 7.20)	5.80 (5.00, 6.90)	6.70 (5.70, 8.32)	0.005
Creatinine (μmol/L)	67 (58, 83)	65 (58, 78)	70.5 (56.8, 93.5)	0.102
Uric acid (μmol/L)	326 (255, 403)	310 (245, 384)	356 (280, 428)	0.019
Na+ (mmol/L)	142 (140, 143)	141 (140, 143)	142(140, 143)	0.807
K+ (mmol/L)	4.05 (3.90, 4.30)	4.00 (3.90, 4.30)	4.10 (3.80, 4.40)	0.251
NT‐proBNP (pg/mL)	111 (41, 347)	66 (23, 131)	533 (239, 1313)	< 0.001
TBil (μmol/L)	9.55 (7.47, 13.93)	9.75 (7.57, 14.00)	9.10 (7.00, 13.93)	0.619
DBil (μmol/L)	4.10 (3.20, 5.53)	4.00 (3.20, 5.32)	4.30 (3.00, 6.12)	0.575
ALT (U/L)	18.5 (13, 26)	19 (13.8, 26.0)	16(11, 24.3)	0.057
AST (U/L)	20.5 (17.0, 26.3)	21.0 (17.0, 27.0)	19.5 (17.0, 25.3)	0.290
HbAlc (%)	6.00 (5.70, 6.60)	6.00 (5.70, 6.60)	6.00 (5.70, 6.66)	0.798
Hs‐Tsh (mU/L)	1.99 (1.38, 2.78)	1.97 (1.31, 2.76)	2.05 (1.56, 2.78)	0.424
Antihypertensive drugs ≥ 2	103 (50.49)	54 (39.71)	49 (72.06)	< 0.001
Diuretic, *n* (%)	34 (16.67)	12 (8.82)	22 (32.35)	< 0.001
MRA, *n* (%)	13 (6.37)	4 (2.94)	9 (13.24)	0.011
Β‐Blocker, *n* (%)	92 (45.10)	57 (41.91)	35 (51.47)	0.196
ACEi/ARB/ARNI, *n* (%)	108 (52.94)	67 (49.26)	41 (60.29)	0.137
SGLT2i, *n* (%)	43 (21.08)	27 (19.85)	16 (23.53)	0.544
PASP (mmHg)	25.0 (21.0, 30.0)	23.0 (20.0, 27.3)	30.0 (24.0, 37.3)	< 0.001
TRPV (m/s)	2.31 (2.13, 2.51)	2.24 (2.08, 2.44)	2.46 (2.30, 2.70)	< 0.001
*E* (cm/s)	70.0 (57.7, 87.3)	66.00(55.8, 79.0)	84.50(66.8, 103.0)	< 0.001
LVEF (%)	65 (62, 67)	65 (63, 67)	63 (59, 66)	0.011
Septal *e*′ (cm/s)	5.50 (4.60, 6.82)	5.75 (4.70, 7.00)	5.10 (4.38, 6.40)	0.046
Lateral *e*′ (cm/s)	7.90 (6.60, 9.20)	8.05 (6.77, 9.40)	7.40 (6.20, 8.53)	0.081
*E*/*e*′ (cm/s)	11.24 ± 3.73	10.11 ± 2.87	13.50 ± 4.23	< 0.001
LAVI (mL/m^2^)	33.52 ± 12.24	28.78 ± 8.66	43.00 ± 12.87	< 0.001
RWT (%)	0.47 ± 0.06	0.47 ± 0.06	0.47 ± 0.07	0.554
LVWT (mm)	11 (10, 11)	10(10, 11)	11(10, 11)	0.431
GLS (%)	18.5 (16.8, 20.0)	18.8 (17.6, 20.2)	17.3 (15.1, 19.4)	< 0.001
RR (s)	0.88 ± 0.15	0.89 ± 0.12	0.85 ± 0.19	0.115
D/S, mean ± SD	1.62 ± 0.31	1.74 ± 0.24	1.39 ± 0.29	< 0.001
LVSTc (%)	38.36 ± 4.85	36.63 ± 3.49	41.81 ± 5.33	< 0.001
QS1 (ms)	0.06 ± 0.02	0.05 ± 0.01	0.07 ± 0.02	< 0.001
QM1 (ms)	95.57 ± 16.94	90.60 ± 12.43	105.51 ± 20.19	< 0.001
EMATc (%)	11.13 ± 3.13	10.28 ± 2.26	12.85 ± 3.86	< 0.001
QS2% (%)	45.48 ± 6.00	44.19 ± 4.89	48.06 ± 7.13	< 0.001
QA2% (%)	48.95 ± 6.81	47.39 ± 5.13	52.07 ± 8.54	< 0.001

*Notes:* Data are %, n (%), mean (SD), or median (IQR), unless indicated otherwise.

Abbreviations: AF, atrial fibrillation; BMI, body mass index; BP, blood pressure; CHD, coronary heart disease; Dbil, direct bilirubin; D/S, the ratio of diastole to systole duration; *E*/*e*′, average septal‐lateral *E*/*e*′ ratio; GLS, global longitudinal strain; hs‐TSH, high‐sensitivity TSH; LAVI, left atrial volume index; LVSTc, left ventricular systolic time/RR; PASP, pulmonary artery systolic pressure; QM1, from the onset of the Q‐wave to the closure of the mitral valve; QS1, from the onset of the Q‐wave to the onset of S1; QS2c, QS2/RR. QA2c, QA2/RR; RWT, relative wall thickness; TBil, total bilirubin; TRPV, tricuspid annular plane systolic excursion.

### 3.2 Variable Selection and Nomogram Construction

**Figure 1:**
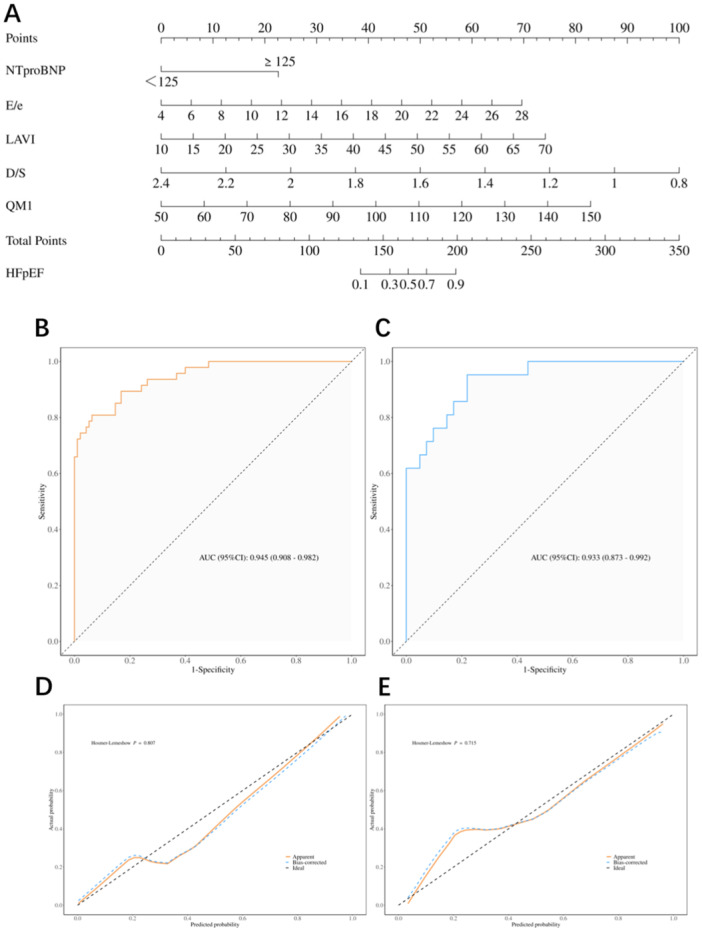
The model for the diagnosis of HFpEF. (A) Nomogram. (B, C) The ROC curves of the nomogram in the training set and the testing set, respectively. (D, E) The calibration curves of the training set and the testing set.

A random stratified sampling was performed, with a ratio of 7:3 between the training set and the testing set (see Supporting Information S1: Table S1). One hundred and forty‐two of the patients were randomly allocated to the training set, while the remaining 62 were assigned to the testing set through random assignment. LASSO regression was applied to analyze the optimal predictive factors in the training set (see Supporting Information S1: Figure S5), and it identified the following as the most significant predictors such as AF, NT‐proBNP, PASP, *E*/*e*′, LAVI, D/S, LVSTc, QM1. Subsequently, multivariable logistic regression analysis was used to construct nomogram using the five most influential factors, including NT‐proBNP (OR = 4.689, 95% CI = 1.352–16.263, *p* = 0.015), *E*/*e*′ (OR = 1.219, 95% CI = 1.017–1.461, *p* = 0.032), LAVI (OR = 1.088, 95% CI = 1.027–1.153, *p* < 0.01), D/S (OR = 0.014, 95% CI = 0.001–0.157, *p* < 0.01), and QM1 (OR = 1.058, 95% CI = 1.02–1.098, *p* < 0.01), as shown in Figure 1A. Among these factors, only D/S was identified as a protective factor for HFpEF (Table 2). The Box–Tidwell test confirmed that the continuous variables met the linearity assumption for binary logistic regression, and all variables showed no collinearity (see Supporting Information S1: Table S2).

**Table 2:** The result of the multivariate logistic analysis.

Variables	Beta	SE	*Z*	*p*	OR (95% CI)
*E*/*e*′	0.20	0.09	2.15	0.032	1.22 (1.02–1.46)
LAVI	0.08	0.03	2.87	0.004	1.09 (1.03–1.15)
D/S	−4.27	1.23	−3.46	< 0.001	0.01 (0.00–0.16)
QM1	0.06	0.02	3.00	0.003	1.06 (1.02–1.10)
NT‐proBNP ≥ 125	1.55	0.63	2.44	0.015	4.69 (1.35–16.26)

*Note:* NT‐proBNP is divided into two groups based on 125 pg/mL.

### 3.3 Nomogram Performance Verification and Evaluation

To evaluate the performance of the nomogram and enhance the model's robustness and generalizability, the data set was repeatedly divided 100 times for training and testing. The average value of each performance metric in the testing set was calculated to represent the model's performance, including AUC, accuracy, sensitivity, specificity, positive predictive value (PPV), and negative predictive value (NPV). Figure 1B,C shows the ROC curve of the nomogram, and the results showed that this model achieved an AUC of 0.933 (95% CI = 0.873–0.992), an accuracy of 0.881, a sensitivity of 0.908, a specificity of 0.844, a PPV of 0.926, and an NPV of 0.823 in the testing set. This indicates that the constructed nomogram has good performance in distinguishing HFpEF. The calibration curve of the model is shown in Figure 1D,E, and the Hosmer–Lemeshow test revealed no statistically significant difference between the predicted and actual clinical probabilities (training set *p* = 0.807 > 0.05, testing set *p* = 0.715 > 0.05), suggesting that the nomogram is well‐calibrated and exhibits good goodness‐of‐fit.

### 3.4 Clinical Efficacy Evaluation of the Nomogram

**Figure 2:**
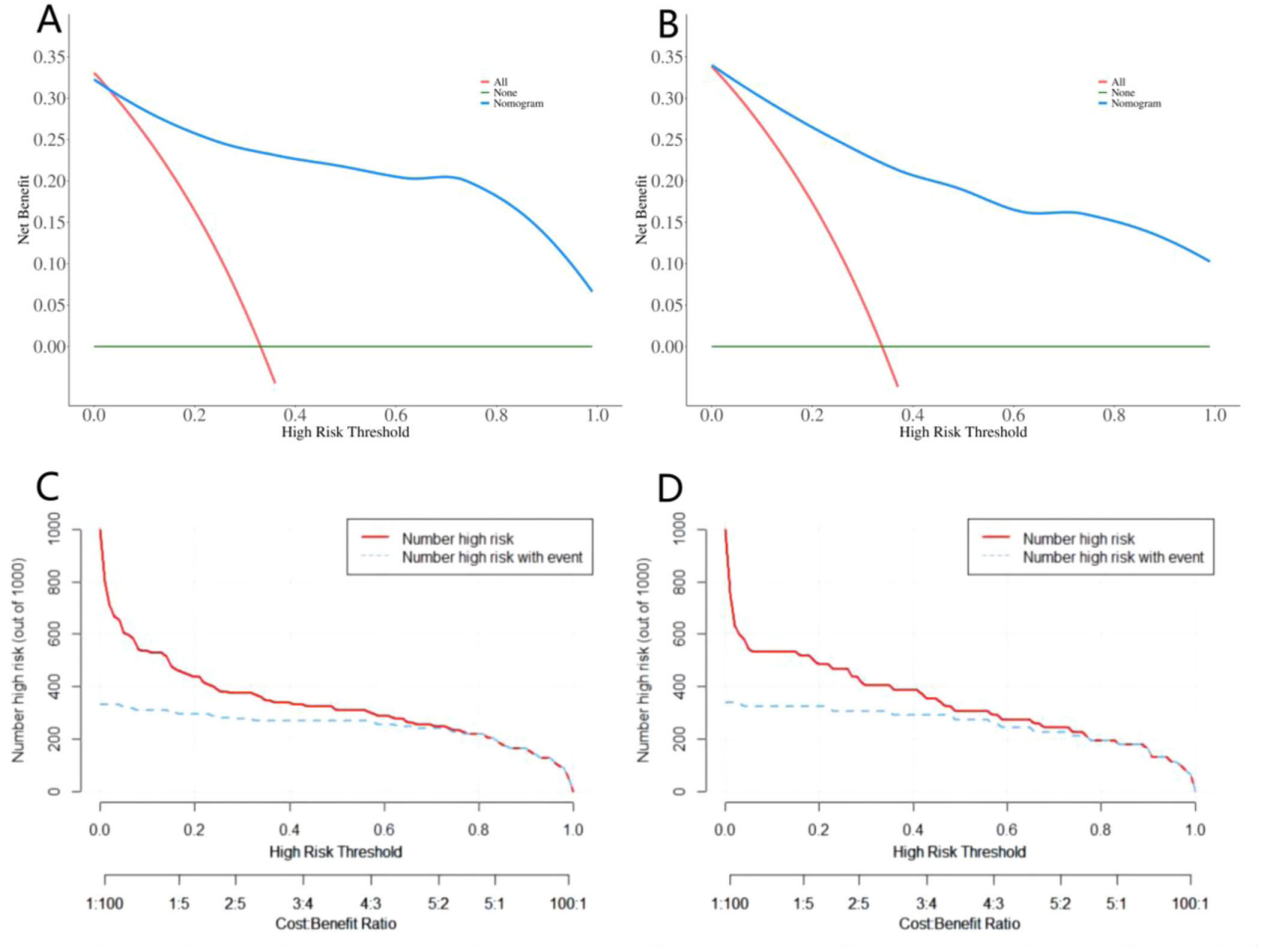
The clinical decision curve analysis of nomogram in (A) the training set and (B) the testing set. The clinical impact curve analysis of nomogram in (C) the training set and (D) the testing set.

The clinical efficacy of the model was analyzed using the DCA and CIC. The outcomes of DCA revealed that both the training set and the testing set achieved maximum benefits at threshold probability values ranging from 0 to 1, indicating good clinical efficacy within this range. Additionally, the CIC analysis demonstrated the high clinical utility of this predictive model. When the threshold probability exceeded 0.5, the model accurately identified individuals at high risk for HFpEF, matching the actual occurrence of HFpEF cases (Figure 2A–D). These findings further confirmed the clinical effectiveness of nomogram.

### 3.5 Comparison of Model Performance and Validation of the Effectiveness of PCG Features

**Figure 3:**
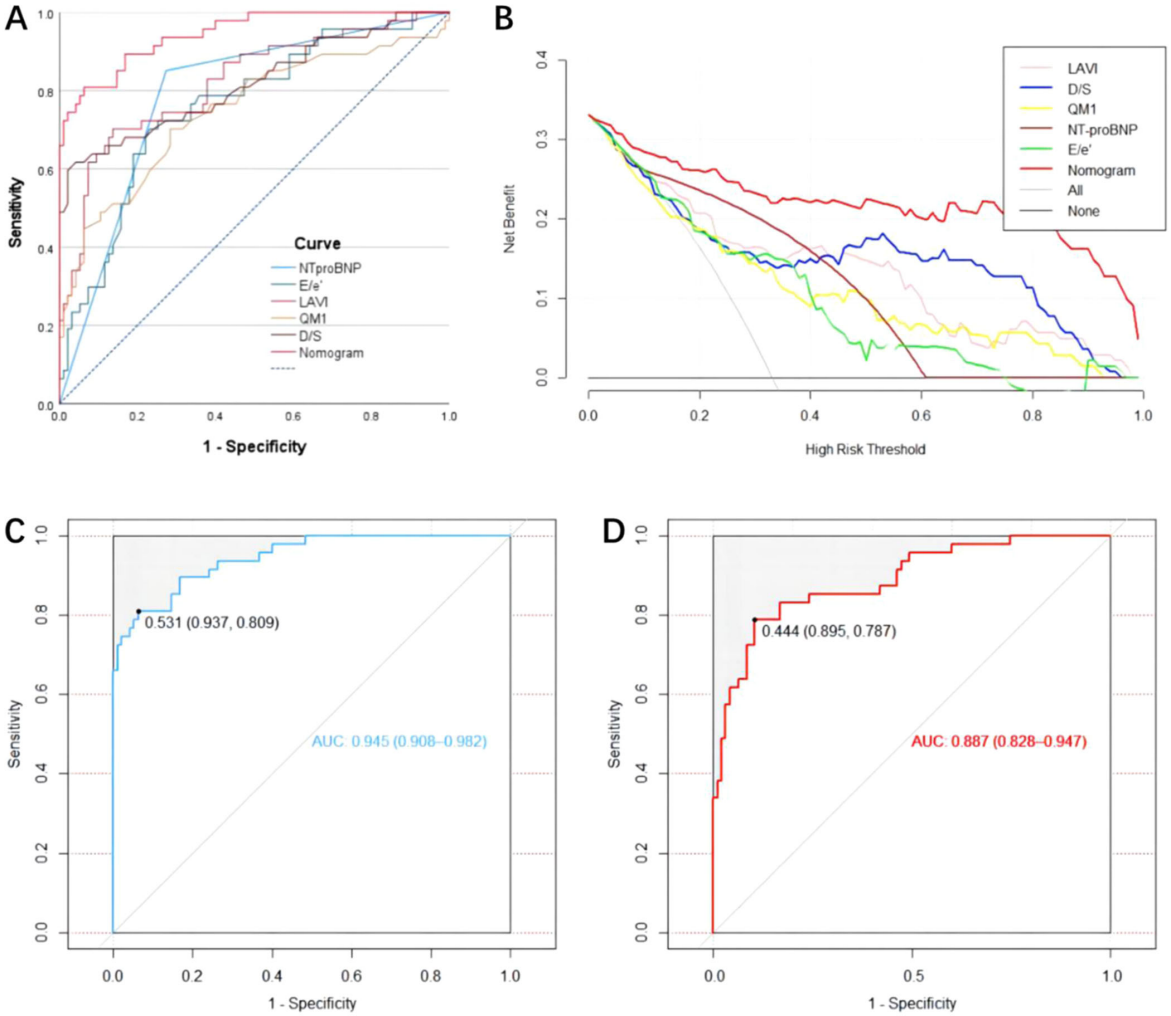
Model comparison. (A) ROC curves of nomogram and models with individual variable for predicting HFpEF. (B) Decision curve analyses comparing the net benefit of the nomogram versus models with individual variable. (C) The ROC of the nomogram. (D) The ROC of the simplified nomogram constructed using *E*/*e*′, LAVI, and NT‐proBNP.

Using logistic regression, a discriminative model was constructed for each screened variable, and the performance was compared with that of a nomogram. As shown in the Figure 3A, the nomogram for HFpEF exhibited significantly higher discriminatory accuracy in predicting HFpEF patients than individual variables incorporated into the nomogram (*p *< 0.001). Moreover, DCAs were employed to evaluate the clinical usefulness of these models. The analyses demonstrated that the nomogram yielded greater net benefit overall compared to models based solely on risk factors included in the nomogram across a wide range of threshold probabilities, as shown in Figure 3B.

Currently, the diagnosis of HFpEF primarily relies on a comprehensive assessment that integrates clinical manifestations, echocardiographic parameters such as *E*/*e*′ and LAVI, and serological biomarkers such as NT‐proBNP. These three diagnostic indicators have been widely applied in clinical practice and is considered a relatively accurate and noninvasive diagnostic strategy. In this study, the proposed nomogram was constructed using these three features such as *E*/*e*′, LAVI, and NT‐proBNP and two PCG features such as D/S and QM1. To verify whether a simplified model built with these three features alone is comparable in performance to the proposed nomogram and to validate the effectiveness of the PCG features. We constructed a simplified nomogram using *E*/*e*′, LAVI, and NT‐proBNP and compared it with the proposed nomogram model. The results show that the AUC for the nomogram model is 0.945 (95% CI = 0.908–0.982), while the AUC for the simplified version is only 0.887 (95% CI = 0.828–0.947), as shown in Figure 3C,D. The DeLong test reveals a statistically significant difference in the AUCs of the two models (*p* < 0.01), demonstrating that the nomogram incorporating PCG features can improve the diagnostic performance of HFpEF based on five features, with an increase in AUC of 0.058. The NRI and IDI results indicated that the proposed nomogram had significantly better prediction performance than the simplified one (NRI = 1.004, 95% CI = 0.697–1.311, *p* < 0.01; IDI = 0.167, 95% CI = 0.091–0.243, *p* < 0.01). This confirmed that the nomogram incorporating PCG features can enhance the diagnostic efficiency of the simplified nomogram for HFpEF in clinical practice.

## 4 Discussion

HFpEF is a highly heterogeneous group of diseases. Rapid and accurate identification of such patients has become a focal point for clinical experts. Currently, the emergence of diagnostic scoring systems like H2FPEF and HFA‐PEFF has facilitated the diagnosis of HFpEF in clinical practice. However, in certain patient populations, their sensitivity or specificity is limited. To address these issues, our study developed a diagnostic model for predicting HFpEF by integrating clinical data, laboratory tests, various medical imaging and signal features. We demonstrate that incorporating PCG and echocardiographic features can effectively enhance the diagnostic performance of HFpEF.

Vermond et al. [33] have found that both HFpEF and atrial fibrillation are linked not only with advanced age, hypertension, and diastolic dysfunction but also with adverse cardiovascular outcomes. Our findings are consistent with this study, revealing significant differences in gender, age, and the occurrence rates of atrial fibrillation and hypertension between patients with HFpEF and the healthy [34]. Although atrial fibrillation is one of the risk factors for HFpEF, it made a lesser contribution to the model and was excluded to avoid overfitting in this study, comparing other studies [19, 20].

In good consistency with other studies [19, 20, 35, 36, 37, 38, 39], *E*/*e*′ and TRPV hold significant value in the prediction of HFpEF. These parameters are commonly used to assess abnormal diastolic function in patients. The *E*/*e*′ ratio shows limited sensitivity in the intermediate range of 9–14. However, when *E*/*e*′ ≥ 15 and TRPV > 3.4 m/s, the presence of diastolic dysfunction can be considered [40]. An *E*/*e*' ratio ≥ 15 demonstrates high specificity in diagnosing HFpEF, while its sensitivity is relatively low [19, 41]. In one cohort where HFpEF prevalence was 64% determined by invasive measurements, the sensitivity and specificity of an *E*/*e*′ ratio > 9 were 78% and 59%, respectively, compared to 46% sensitivity and 86% specificity for *E*/*e*′ > 13 [19]. Similarly, in our study, the sensitivity and specificity of an *E*/*e*′ ratio > 9 were 82% and 51%, respectively, compared to 47% sensitivity and 87% specificity for *E*/*e*′ > 13. Notably, an *E*/*e*′ ratio ≥ 15 in our study exhibited a sensitivity of 24% and a specificity of 96%.

The LAVI is an indirect correlate of LV filling pressures [42] and serves as a more accurate marker of chronic LA remodeling compared to LA area or diameter [43, 44, 45]. Multiple studies have demonstrated that an LAVI > 34 mL/m^2^ can independently predict HF, atrial fibrillation, and ischemic stroke events [46, 47, 48]. In our study, the sensitivity and specificity of an LAVI > 34 mL/m^2^ for diagnosing HFpEF were 72% and 71%, respectively. Importantly, the findings suggest that using *E*/*e*′ or LAVI alone for diagnosing HFpEF does not yield superior performance. Therefore, this study combines echocardiographic functional and morphological indictors such as *E*/*e*′ and LAVI to accurately identify the patients with HFpEF.

D/S is a crucial indicator for assessing cardiac diastolic reserve, and evaluating whether the heart has sufficient self‐supply time. In the patients with HFpEF, left ventricular diastolic function is impaired, leading to a shortened diastolic period. A decreased D/S reflects this functional impairment. During diastole, the heart replenishes its blood supply; a prolonged diastolic period enhances ventricular blood supply, providing more nutrients and oxygen for systole, thus strengthening myocardial contractility. Conversely, insufficient diastole results in deficient ventricular filling, decreased cardiac output, reduced self‐blood supply, diminished myocardial contractility, and this can even lead to ischemic myocardial injury or cardiac death [49, 50]. These findings align with those observed in earlier studies, revealing a significant decrease in the D/S among patients with HFpEF compared to non‐HFpEF. In this study, the D/S was not inferior to LAVI in identifying HFpEF, with AUC values of 0.816 and 0.815, respectively. A cut‐off value of 1.43 provided 93% specificity but only 62% sensitivity. This indicates that the D/S has high accuracy in screening and excluding low‐risk individuals for HFpEF, with a low risk of misdiagnosis.

A study evaluating the discharge outcomes of 45 hospitalized patients with acute HF confirmed that EMAT (QM1) is an independent risk factor for cardiovascular prognosis [51]. In clinical practice, an EMAT value > 120 ms [52] or EMATc > 15% [53] is considered an indicator of reduced cardiac systolic function. Previous research on EMAT has primarily focused on patients with LVEF < 50%, and there is currently a lack of research specifically related to HFpEF. This study differs from previous researches by revealing a significant prolongation of QM1 in patients with HFpEF compared to non‐HFpEF (*p* < 0.01). Surprisingly, the diagnostic performance of *E*/*e*′ (AUC 0.762) was not superior to that of QM1 (AUC 0.731) for diagnosing HFpEF, as indicated by the Delong test showing no statistically significant difference (*p* > 0.05). With an optimal cut‐off value of 105 ms, QM1 specificity increased to 89%, demonstrating a high negative predictive value. QM1 represents the interval from ventricular electrical activity to mechanical activity, reflecting the time required for the ventricle to generate sufficient pressure for mitral valve closure (left ventricular pressure > left atrial pressure).

The discordant changes in left atrial pressure and left ventricular pressure can lead to an extension of QM1. A plausible explanation is that the elevation of left atrial pressure, resulting from impaired diastolic function in HFpEF, necessitates higher pressures for normal blood filling. Additionally, increased ventricular stiffness may prolong the time required for ventricular pressure to achieve mitral valve closure. While the integration of heart sound features requires additional equipment and analysis, it offers a valuable diagnostic and management, ultimately enhancing overall medical quality and patient care effectiveness.

In contrast, there are more researches on natriuretic peptides in the diagnosis of HFpEF, with diagnostic thresholds varying depending on the presence of atrial fibrillation [17, 54, 55]. One study demonstrated that NT‐proBNP ≥ 125 pg/mL had a sensitivity of 85% and a specificity of 88% for diagnosing HFpEF [32]. Reports indicate that during screening, the average NT‐proBNP levels in patients with atrial fibrillation were found to be 3–3.5 times higher compared to patients with sinus rhythm [56, 57, 58, 59]. Therefore, when diagnosing HFpEF, it is generally recommended to set the threshold for patients with atrial fibrillation at three times higher than for patients with sinus rhythm (NT‐proBNP ≥ 375 pg/mL) [20]. To simplify the model for clinical application, based on previous literature and guidelines, this study classified NT‐proBNP with a cut‐off value of 125 pg/mL. In this study, NT‐proBNP ≥ 125 pg/mL demonstrated a sensitivity of 85% and a specificity of 73%. Incorporating NT‐proBNP into the model offers several benefits: it is scientifically validated by international guidelines for its diagnostic and prognostic roles [60, 61]; it is cost‐effective and noninvasive, facilitating widespread clinical application; and it enables risk stratification for tailored patient management and improved outcomes.

This study adopted LASSO and logistic regression analysis to confirm the independent risk factors of HFpEF and construct a nomogram, including two PCG features such as D/S and QM1, two echocardiographic features such as *E*/*e*′ and LAVI, and a commonly used clinical indicator NT‐proBNP. Through the above analysis, it is evident that each feature contributes to the diagnosis of HFpEF and is an independent predictor of HFpEF. This study combined these five features to construct a comprehensive nomogram model. By integrating the advantages of each indicator, the comprehensive model outperformed models built with single indicators in diagnosing HFpEF. The nomogram quantified and visualized model outcomes, facilitating the estimation of independent variables and personalized HFpEF risk prediction, enhancing physician understanding and acceptance. We also compared the performance of a simplified nomogram constructed using *E*/*e*′, LAVI, and NT‐proBNP with the proposed nomogram model. The results showed that incorporating PCG features enhanced the model's performance in diagnosing HFpEF, and NRI and IDI analyses also indicated that the proposed nomogram had significantly better prediction performance, demonstrating the effectiveness of PCG features. The model's high diagnostic accuracy facilitates the precise identification of HFpEF patients, enabling timely and guideline‐specific management by physicians. Early diagnosis and intervention may reduce HF hospitalization rates, lower complication incidence, and are anticipated to extend patient survival.

There are several limitations in this study to be addressed. First, this study is a single‐center cross‐sectional study with a small sample size. Next, large‐sample data should be included to further increase the model's generalizability, and external validation data from multiple institutions should be incorporated to verify the model's effectiveness. Second, the study predominantly focuses on diagnostic accuracy without extensive further investigation into the long‐term clinical implications of the model, such as how the model influences patient outcomes. Future research should include longitudinal studies to assess the impact of this diagnostic tool on clinical outcomes. Third, some HFpEF patients experienced short‐term readmission, and their prior medication treatment might have influenced PCG characteristics, potentially impacting our results, which is challenging to control. Lastly, due to the limited sample size, we did not perform subgroup analysis. Further prospective studies should conduct subgroup analyses to evaluate and validate the diagnostic effectiveness of the constructed nomogram across different subgroups. Future studies should conduct large‐sample multi‐center clinical research and subgroup analysis to optimize and externally validate the model. Additionally, it is particularly emphasized that assessing the impact of this diagnostic tool on clinical outcomes through longitudinal studies is crucial, positioning it as a significant direction for future research.

## 5 Conclusions

In conclusion, this study presents a comprehensive nomogram integrating phonocardiogram and echocardiogram features for the noninvasive diagnosis of HFpEF. The nomogram was constructed using machine learning to select three variables from routine clinical indicators, combined with two heart sound features, including *E*/*e*', LAVI, NT‐proBNP, D/S, and QM1. The results show that the nomogram, built with five easily obtainable clinical indicators, outperforms the simplified version in diagnosing HFpEF. Additionally, the nomogram can visualize model outputs and quantify individual event risk ratios. We also identified that D/S and QM1 are independent risk factors for HFpEF and can be used as supplementary cardiovascular biomarkers. The findings of this study indicate that a nomogram based on phonocardiogram and echocardiogram features can assist in HFpEF diagnosis, providing a valuable reference for early clinical diagnosis.

## Conflicts of Interest

The authors declare no conflicts of interest.

## Supporting information

Supporting information.

## Data Availability

Our Expects Data Policy requires a Data Availability Statement, even if no data are available, so please enter one in the space below. Sample statements can be found here. Please note that this statement will be published alongside your manuscript, if it is accepted for publication. The data that support the findings of this study are available on request from the corresponding author. The data are not publicly available due to privacy or ethical restrictions.
