# Supplementary material for: A Comprehensive Nomogram Integrating Phonocardiogram and Echocardiogram Features for the Diagnosis of Heart Failure With Preserved Ejection Fraction
Source: Clin Cardiol. 2024 Oct 28;47(11):e70022. doi: 10.1002/clc.70022 (PMC11514106; doi:10.1002/clc.70022)
Supplement: Supplementary file 1 — Supporting information. [file CLC-47-e70022-s001.docx]

ONLINE SUPPLEMENTS FOR

# A comprehensive nomogram integrating phonocardiogram and echocardiogram features for the diagnosis of heart failure with preserved ejection fraction

Linchun Cao^2,6^, Xingming Guo^3^, Kangla Liao^2^, Jian Qin^2^, Yineng Zheng^1,4,5^*****

1 Department of Radiology, The First Affiliated Hospital of Chongqing Medical University, Chongqing 400016, PR China.

2 Department of Cardiology, The First Affiliated Hospital of Chongqing Medical University, Chongqing 400016, PR China.

3 Key Laboratory of Biorheology Science and Technology, Ministry of Education, College of Bioengineering, Chongqing University, Chongqing 400044, PR China.

4 State Key Laboratory of Ultrasound in Medicine and Engineering, Chongqing Medical University, Chongqing, 400016, China.

5 Medical Data Science Academy, Chongqing Medical University, Chongqing, 400016, China.

6.Department of Cardiology, People's Hospital of Fengjie County, Chongqing 404600, PR China.

Address for Correspondence:

***** correspondence to: Yineng Zheng

E-mail: yinengzheng@cqmu.edu.cn

Address: No. 1 Youyi Road, Yuzhong District, Chongqing 400016, People’s Republic of China.

**Table of Contents**

P3: Supplementary Table S1: TRIPOD checklist for prediction model development and validation.

P5: Supplementary Methods: Diagnosis of heart failure with preserved ejection fraction.

P6: Supplementary Figure S1: The flowchart of inclusion and exclusion.

P7: Supplementary Figure S2: Heart sound and Electrocardiogram.

P9: Supplementary Figure S3: Measuring the average septal-lateral E/e′ratio.

P9: Supplementary Figure S4: Measuring Left atrial volume index.

P10: Supplementary Table S1: Comparison of features between the training and test sets.

P13: Supplementary Figure S5: LASSO result.

P14: Supplementary Table S2: Collinearity test.

P14: Supplementary RF: A sensitivity analysis by using the random forest (RF) algorithm in the original data.

P15: Supplementary References.

**Supplementary Table**

**Supplementary Table S1:** TRIPOD checklist for prediction model development and validation

| **Section/Topic** | **Item** |  | **Checklist Item** | **Page** |
| --- | --- | --- | --- | --- |
| **Title and abstract** |  |  |  |  |
| Title | 1 | D;V | Identify the study as developing and/or validating a multivariable prediction model, the target population, and the outcome to be predicted. | 1 |
| Abstract | 2 | D;V | Provide a summary of objectives, study design, setting, participants, sample size, predictors, outcome, statistical analysis, results, and conclusions. | 1 |
| **Introduction** |  |  |  |  |
| Background and objectives | 3a | D;V | Explain the medical context (including whether diagnostic or prognostic) and rationale for developing or validating the multivariable prediction model, including references to existing models. | 1 |
|  | 3b | D;V | Specify the objectives, including whether the study describes the development or validation of the model or both. | 3 |
| **Methods** |  |  |  |  |
| Source of data | 4a | D;V | Describe the study design or source of data (e.g., randomized trial, cohort, or registry data), separately for the development and validation data sets, if applicable. | 4 |
|  | 4b | D;V | Specify the key study dates, including start of accrual; end of accrual; and, if applicable, end of follow-up. | 4 |
| Participants | 5a | D;V | Specify key elements of the study setting (e.g., primary care, secondary care, general population) including number and location of centres. | 4 |
|  | 5b | D;V | Describe eligibility criteria for participants. | 4 |
|  | 5c | D;V | Give details of treatments received, if relevant. |  |
| Outcome | 6a | D;V | Clearly define the outcome that is predicted by the prediction model, including how and when assessed. | 5 |
|  | 6b | D;V | Report any actions to blind assessment of the outcome to be predicted. | 5 |
| Predictors | 7a | D;V | Clearly define all predictors used in developing or validating the multivariable prediction model, including how and when they were measured. | 5 |
|  | 7b | D;V | Report any actions to blind assessment of predictors for the outcome and other predictors. | 5 |
| Sample size | 8 | D;V | Explain how the study size was arrived at. | 5 |
| Missing data | 9 | D;V | Describe how missing data were handled (e.g., complete-case analysis, single imputation, multiple imputation) with details of any imputation method. | 6 |
| Statistical analysis methods | 10a | D | Describe how predictors were handled in the analyses. | 6 |
|  | 10b | D | Specify type of model, all model-building procedures (including any predictor selection), and method for internal validation. | 6 |
|  | 10c | V | For validation, describe how the predictions were calculated. | 6 |
|  | 10d | D;V | Specify all measures used to assess model performance and, if relevant, to compare multiple models. | 6 |
|  | 10e | V | Describe any model updating (e.g., recalibration) arising from the validation, if done. |  |
| Risk groups | 11 | D;V | Provide details on how risk groups were created, if done. |  |
| Development vs. validation | 12 | V | For validation, identify any differences from the development data in setting, eligibility criteria, outcome, and predictors. | 6 |
| **Results** |  |  |  |  |
| Participants | 13a | D;V | Describe the flow of participants through the study, including the number of participants with and without the outcome and, if applicable, a summary of the follow-up time. A diagram may be helpful. | 7 |
|  | 13b | D;V | Describe the characteristics of the participants (basic demographics, clinical features, available predictors), including the number of participants with missing data for predictors and outcome. | 7 |
|  | 13c | V | For validation, show a comparison with the development data of the distribution of important variables (demographics, predictors and outcome). | 7 |
| Model development | 14a | D | Specify the number of participants and outcome events in each analysis. | 7 |
|  | 14b | D | If done, report the unadjusted association between each candidate predictor and outcome. | 7 |
| Model specification | 15a | D | Present the full prediction model to allow predictions for individuals (i.e., all regression coefficients, and model intercept or baseline survival at a given time point). | 7 |
|  | 15b | D | Explain how to the use the prediction model. | 8 |
| Model performance | 16 | D;V | Report performance measures (with CIs) for the prediction model. | 8 |
| Model-updating | 17 | V | If done, report the results from any model updating (i.e., model specification, model performance). | - |
| **Discussion** |  |  |  |  |
| Limitations | 18 | D;V | Discuss any limitations of the study (such as nonrepresentative sample, few events per predictor, missing data). | 12 |
| Interpretation | 19a | V | For validation, discuss the results with reference to performance in the development data, and any other validation data. | 8 |
|  | 19b | D;V | Give an overall interpretation of the results, considering objectives, limitations, results from similar studies, and other relevant evidence. | 9 |
| Implications | 20 | D;V | Discuss the potential clinical use of the model and implications for future research. | 12 |
| **Other information** |  |  |  |  |
| Supplementary information | 21 | D;V | Provide information about the availability of supplementary resources, such as study protocol, Web calculator, and data sets. | - |
| Funding | 22 | D;V | Give the source of funding and the role of the funders for the present study. | 13 |

**Supplementary Methods**

**Diagnosis of heart failure with preserved ejection fraction**

The high-quality images will be stored for all patients who will to participate in the study, and the relevant parameters needed for both the H_2_FPEF and HFA-PEFF scores were calculated [1-3]. A H_2_FPEF score ≥6 or HFA-PEFF score ≥5 was used as a rule‑in criterion for HFpEF. The H2FPEF score relies on 2 echocardiographic parameters (E/e’ >9 and pulmonary artery systolic pressure >35 mmHg, each scoring 1 point) and clinical variables associated with HFpEF, including obesity (2 points), any history of atrial fibrillation (3 points), age >60 years (1 point), and use of ≥2 antihypertensive medications (1 point). By establishing the probability of disease, the H2FPEF score may be used to establish the diagnosis with reasonably high confidence at higher scores (6–9). The hemodynamic definition of HFpEF in this study was a pulmonary arterial wedge pressure ≥15 mmHg at rest or ≥25 mmHg during exercise.

Based on echocardiographic correlates of invasive hemodynamic measurements, the Heart Failure Association of the European Society of Cardiology in consensus, has proposed an alternative score to aid the diagnosis of HFpEF. The HFA‑PEFF score is calculated from echocardiographic measures of cardiac function and morphology in addition to natriuretic peptide levels. Each of those 3 components contributes 2 points to yield a maximal score of 6, with a score ≥5 to confirm HFpEF. This figure only shows the scoring parameters and threshold criteria used in this study.

| H2FPEF and HFA-PEFF score | | |
| --- | --- | --- |
| H2FPEF score （≥6 points rule-in) | | |
| Heavy | Body mass index >30kg/m^2^ | 2 point |
| Hypertensive | Treatment with ≥2 antihypertensives | 1 point |
| Atrial Fibrillation | Paroxysmal or Persistent | 3 point |
| Pulmonary hypertension | PASP >35mmHg | 1 point |
| Elder | Age >60 years | 1 point |
| Filling Pressure | E/e’ratio >9 | 1 point |
| HFA-PEFF score (≥5points rule-in) | | |
| Functional | Morphological | Biomarker |
| Major criterion 2 point | | |
| Septal e′ <7 cm∕s or lateral e′ <10 cm∕s；  Average septal-lateral E∕e′ ratio ≥15；  TR peak velocity >2.8 m∕s；  PASP >35 mmHg | LAVI >34 mL∕m^2^；  LVMI ≥149/122 g∕m^2^ (m/w)  and RWT >0.42 | NT-proBNP >220  pg/ml(SR)；  NT-proBNP >660  pg/ml(AF) |
| Minor criterion 1 point | | |
| E∕e′ ratio 9-14；  GLS <16% | LAVI 29 −34 mL∕m^2^；  LVMI ≥115/95 g∕m^2^ (m/w)  or RWT >0.42；  LV end-diastolic wall thickness ≥12 mm | NT-proBNP 125-220pg/ml(SR)；  NT-proBNP 375-660pg/ml(AF) |

*PASP pulmonary artery systolic pressure; SR sinus rhythm; m/w female/male; AF Atrial fibrillation;*

*LVMI left ventricular mass index; LAVI left atrial volume index; RWT relative wall thickness;*

*TR tricuspid regurgitation; GLS left ventricular global longitudinal systolic strain.*

**Supplementary Figures**

**Supplementary Figure S1: The flowchart of inclusion and exclusion.**


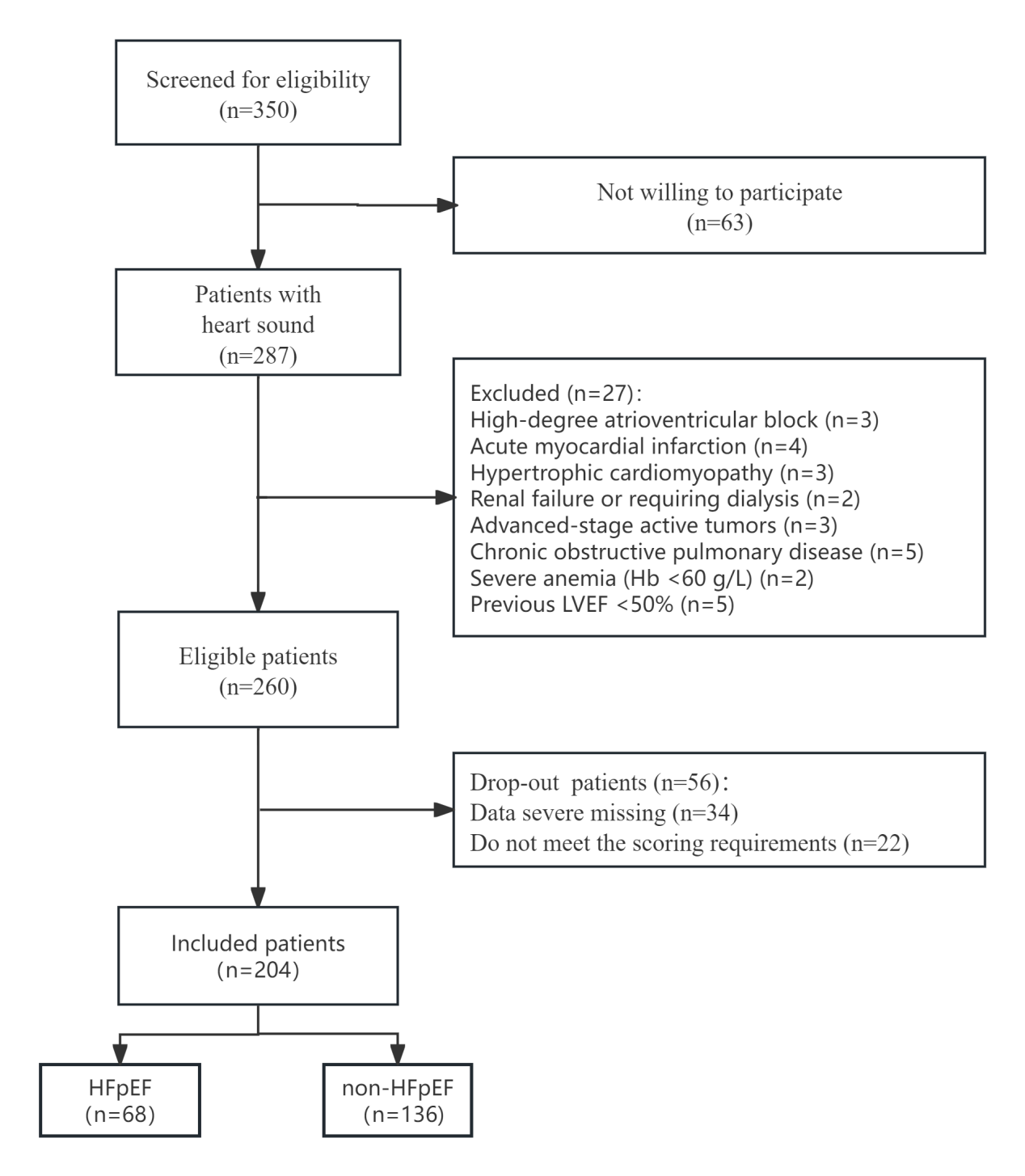


**Supplementary Figure S2: PCG and ECG.**


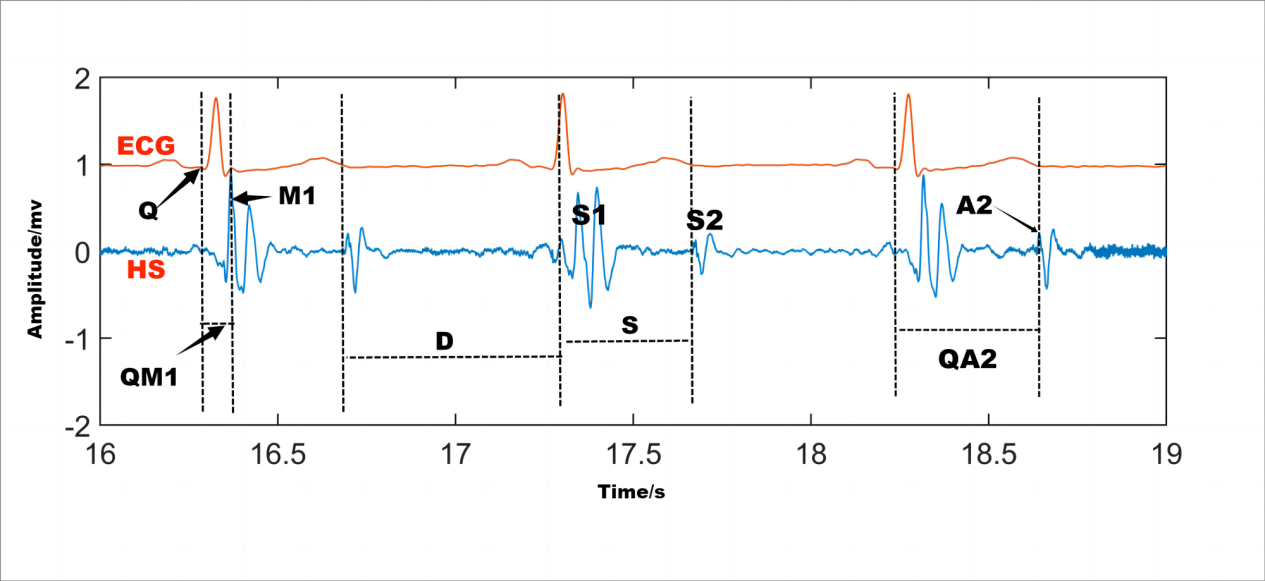


D/S: the ratio of diastole to systole duration. D represents the time interval from the onset of the second heart sound to the onset of the first heart sound in the next cycle, while S represents the time interval from the onset of the first heart sound to the onset of the second heart sound.

QS1: time interval from the onset of the Q-wave on electrocardiography to the first heart sound. Localization of the Q-wave onset was followed by recording its initiation time, which was synchronized with the PCG. Under normal conditions, the S1 onset occurs 40-80 milliseconds after the Q-wave onset, and within this range, an initial screening for the appropriate S1 onset was conducted. M1 is often more discernible than S1. By identifying M1's position and inferring the S1 onset point, given that mitral valve closure producing M1 typically happens 20-40 milliseconds after the S1 onset, the S1 onset point was localized comprehensively through these two steps.

QM1: time interval from the onset of the Q-wave to the closure of the mitral valve. M1 is defined as the first peak of the normal first heart sound, which is produced by the closure of the mitral valve, occurring 0.03-0.05 seconds before the closure of the tricuspid valve.

QA2: time interval from the onset of the Q-wave to the closure of the aortic valve. A2 is defined as the first peak of the second heart sound, which is produced by the closure of the aortic valve, it closes usually slightly earlier than the pulmonary valve.

**Supplementary Figure S3: Measuring the average septal-lateral E/e′ratio.**

**
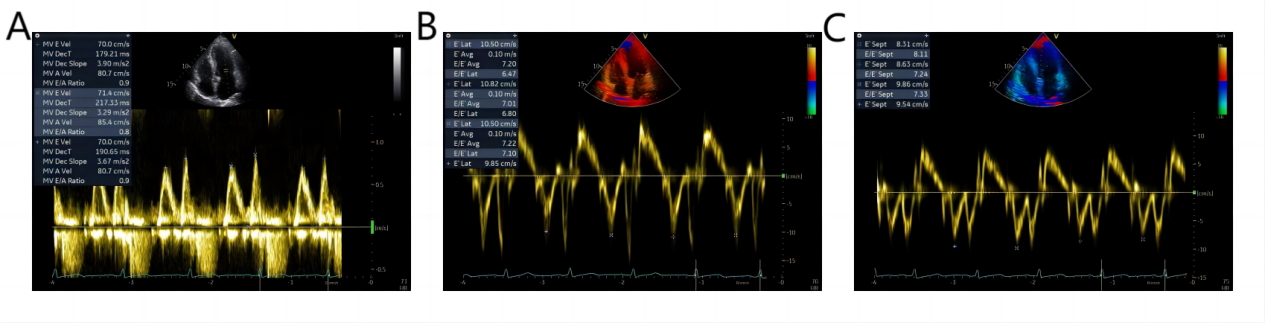
**

**The ratio of the peak velocity of mitral inflow during early diastole (E), recorded by pulsed Doppler between the tips of the mitral leaflets, over the average of septal and lateral mitral annular early diastolic peak velocities (e′) recorded by pulsed tissue Doppler (3).**

**Supplementary Figure S4: Measuring Left atrial volume index.**

**
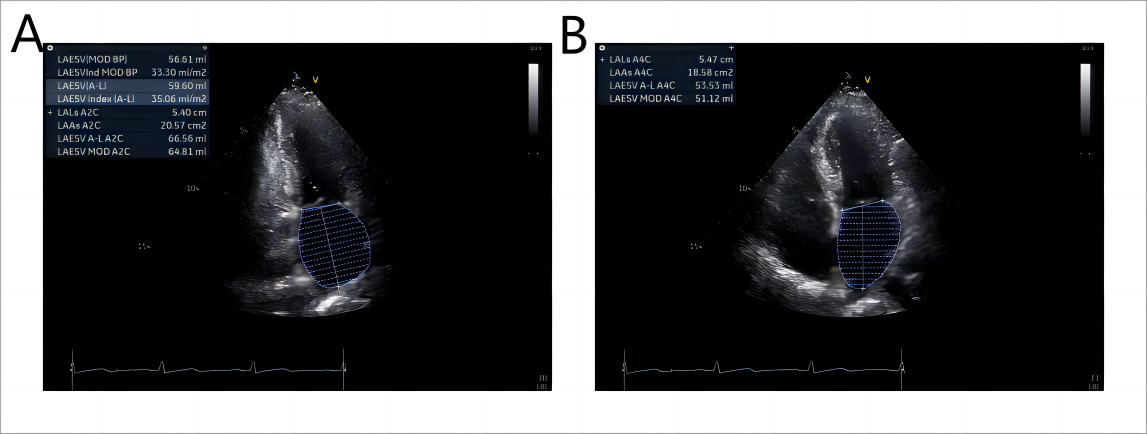
**

**The maximal volume of the LA, measured at end-systole from biplane (3) and indexed to body surface area left atrial volume index (LAVI).**

**Supplementary Table S1: Comparison of features between the training and test sets**

| Variable | Total (n = 204) | Training set (n = 142) | Test set (n = 62) | P |
| --- | --- | --- | --- | --- |
| Age, Mean ± SD | 65.82 ± 11.28 | 66.30 ± 11.43 | 64.73 ± 10.95 | 0.360 |
| Female, n (%) | 99 (48.53) | 67 (47.18) | 32 (51.61) | 0.560 |
| BMI, Mean ± SD | 24.32 ± 3.14 | 24.45 ± 3.06 | 24.05 ± 3.31 | 0.402 |
| Systolic BP, Mean ± SD | 135.42 ± 18.95 | 135.90 ± 18.55 | 134.32 ± 19.95 | 0.585 |
| Hypertension, n (%) | 141 (69.12) | 95 (66.90) | 46 (74.19) | 0.300 |
| Hyperlipidemia, n (%) | 52 (25.49) | 38 (26.76) | 14 (22.58) | 0.529 |
| AF, n (%) | 28 (13.73) | 18 (12.68) | 10 (16.13) | 0.510 |
| T2DM, n (%) | 73 (35.78) | 49 (34.51) | 24 (38.71) | 0.565 |
| CHD, n (%) | 101 (49.51) | 70 (49.30) | 31 (50.00) | 0.926 |
| Stent, n (%) | 41 (20.1) | 29 (20.42) | 12 (19.35) | 0.861 |
| Smoking, n (%) | 59 (28.92) | 43 (30.28) | 16 (25.81) | 0.517 |
| Hb, Mean ± SD | 133.79 ± 19.87 | 134.29 ± 19.79 | 132.65 ± 20.16 | 0.588 |
| BUN, M (Q₁, Q₃) | 6.05 (5.10 - 7.20) | 6.10 (5.10 - 7.20) | 5.90 (5.12 - 7.10) | 0.817 |
| Cr, M (Q₁, Q₃) | 67.00 (58.00 - 83.25) | 69.00 (59.00 - 86.25) | 62.50 (54.00 - 73.75) | 0.005 |
| UA, M (Q₁, Q₃) | 326.00 (254.50 - 403.00) | 338.00 (269.25 - 411.25) | 300.00 (239.25 - 372.50) | 0.111 |
| Na+, M (Q₁, Q₃) | 142.00 (140.00 - 143.00) | 142.00 (140.00 - 143.00) | 141.50 (140.00 - 143.00) | 0.477 |
| K+, M (Q₁, Q₃) | 4.05 (3.90 - 4.30) | 4.05 (3.90 - 4.30) | 4.05 (3.90 - 4.30) | 0.666 |
| NT-proBNP, M (Q₁, Q₃) | 110.50 (40.75 - 347.00) | 104.50 (38.25 - 349.00) | 132.50 (48.75 - 317.00) | 0.576 |
| TBil, M (Q₁, Q₃) | 9.55 (7.47 - 13.93) | 9.20 (7.20 - 13.80) | 10.30 (7.93 - 14.15) | 0.281 |
| DBil, M (Q₁, Q₃) | 4.10 (3.20 - 5.53) | 3.95 (3.20 - 5.30) | 4.55 (3.20 - 6.15) | 0.117 |
| ALT, M (Q₁, Q₃) | 18.50 (13.00 - 26.00) | 19.00 (12.00 - 26.00) | 18.00 (13.00 - 25.00) | 0.816 |
| AST, M (Q₁, Q₃) | 20.50 (17.00 - 26.25) | 21.00 (17.00 - 26.75) | 20.00 (18.00 - 25.75) | 0.907 |
| HbAlc, M (Q₁, Q₃) | 6.00 (5.70 - 6.60) | 6.00 (5.70 - 6.50) | 6.00 (5.70 - 6.77) | 0.247 |
| hs-Tsh, M (Q₁, Q₃) | 1.99 (1.38 - 2.78) | 2.08 (1.37 - 2.82) | 1.90 (1.39 - 2.53) | 0.415 |
| Antihypertensive drugs≥2 | 103 (50.49) | 71 (50.00) | 32 (51.61) | 0.832 |
| Diuretic, n (%) | 34 (16.67) | 20 (14.08) | 14 (22.58) | 0.134 |
| MRA, n (%) | 13 (6.37) | 9 (6.34) | 4 (6.45) | 1.000 |
| Β Blocker, n (%) | 92 (45.1) | 65 (45.77) | 27 (43.55) | 0.769 |
| ACEi/ARB/ARNI, n (%) | 108 (52.94) | 74 (52.11) | 34 (54.84) | 0.720 |
| SGLT2i, n (%) | 43 (21.08) | 29 (20.42) | 14 (22.58) | 0.728 |
| PASP, M (Q₁, Q₃) | 25.00 (21.00 - 30.00) | 25.00 (21.00 - 30.00) | 25.00 (21.00 - 30.00) | 0.988 |
| TRPV, M (Q₁, Q₃) | 2.31 (2.13 - 2.51) | 2.31 (2.13 - 2.50) | 2.33 (2.13 - 2.53) | 0.822 |
| E, M (Q₁, Q₃) | 70.00 (57.67 - 87.25) | 71.50 (61.00 - 88.00) | 69.50 (56.25 - 83.75) | 0.375 |
| LVEF, M (Q₁, Q₃) | 65.00 (62.00 - 67.00) | 65.00 (62.00 - 67.00) | 65.00 (62.00 - 67.00) | 0.565 |
| Septal e’, M (Q₁, Q₃) | 5.50 (4.60 - 6.82) | 5.50 (4.60 - 6.68) | 5.60 (4.53 - 7.07) | 0.462 |
| Lateral e’, M (Q₁, Q₃) | 7.90 (6.60 - 9.20) | 7.80 (6.53 - 9.00) | 8.20 (6.65 - 9.73) | 0.294 |
| E/e’, M (Q₁, Q₃) | 10.81 (8.66 - 12.99) | 11.00 (8.65 - 13.45) | 9.84 (8.69 - 12.33) | 0.180 |
| LAVI, M (Q₁, Q₃) | 31.52 (24.03 - 41.65) | 31.26 (23.86 - 41.85) | 32.15 (24.61 - 40.79) | 0.602 |
| RWT, M (Q₁, Q₃) | 0.47 (0.43 - 0.51) | 0.47 (0.43 - 0.52) | 0.47 (0.43 - 0.49) | 0.351 |
| LVWT, M (Q₁, Q₃) | 11.00 (10.00 - 11.00) | 10.00 (10.00 - 11.00) | 11.00 (10.00 - 11.00) | 0.853 |
| GLS, M (Q₁, Q₃) | 18.50 (16.78,20.02) | 18.60 (17.00,20.10) | 18.05 (16.35,19.95) | 0.230 |
| RR, M (Q₁, Q₃) | 0.87 (0.79 - 0.98) | 0.88 (0.80 - 0.98) | 0.84 (0.74 - 0.96) | 0.069 |
| D/S, M (Q₁, Q₃) | 1.63 (1.43 - 1.83) | 1.64 (1.47 - 1.87) | 1.60 (1.40 - 1.78) | 0.168 |
| LVSTc, M (Q₁, Q₃) | 38.06 (34.73 - 40.96) | 37.81 (34.28 - 40.75) | 38.29 (35.30 - 41.97) | 0.275 |
| QS1, M (Q₁, Q₃) | 0.06 (0.05 - 0.07) | 0.06 (0.05 - 0.07) | 0.05 (0.05 - 0.07) | 0.775 |
| QM1, M (Q₁, Q₃) | 94.00 (84.00 - 105.00) | 94.50 (84.25 - 104.00) | 93.00 (82.50 - 105.75) | 0.856 |
| EMATc, M (Q₁, Q₃) | 10.77 (8.98 - 12.77) | 10.70 (8.84 - 12.84) | 11.17 (9.40 - 12.67) | 0.445 |
| QS2%, M (Q₁, Q₃) | 45.10 (40.92 - 49.02) | 44.69 (40.77 - 48.55) | 45.92 (41.66 - 50.80) | 0.079 |
| QA2%, M (Q₁, Q₃) | 48.10 (43.97 - 52.18) | 47.56 (43.64 - 51.45) | 49.87 (45.14 - 54.44) | 0.036 |

**Supplementary Figure S5:** Best match factor screening process by lasso regression. A is the Lasso regression path diagram; B shows the plot of the best matching factors screened by lasso regression, and the best matching factors were selected using lambda.1se as the criterion (8 variables corresponding to the vertical line on the right).


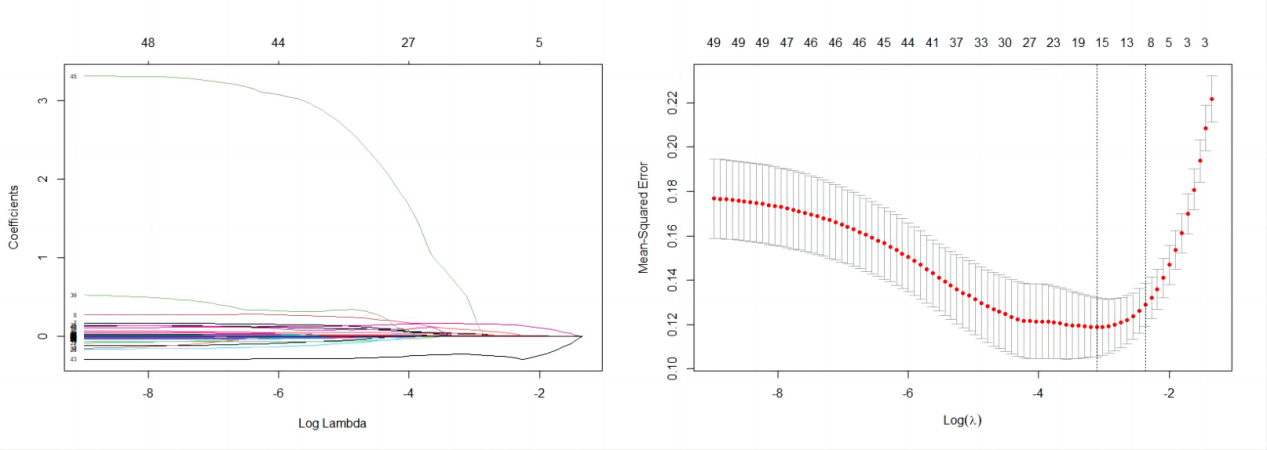


**Supplementary Table S2: Collinearity test**

| Variables | Tol | VIF |
| --- | --- | --- |
| LAVI | 0.661 | 1.513 |
| D/S | 0.789 | 1.268 |
| QM1 | 0.887 | 1.127 |
| NTproBNP | 0.708 | 1.413 |
| E/e’ | 0.773 | 1.294 |

If the tolerance <0.1 or the VIF >5, it indicates the presence of collinearity.

**Supplementary RF: A sensitivity analysis by using the random forest (RF) algorithm in the original data.**


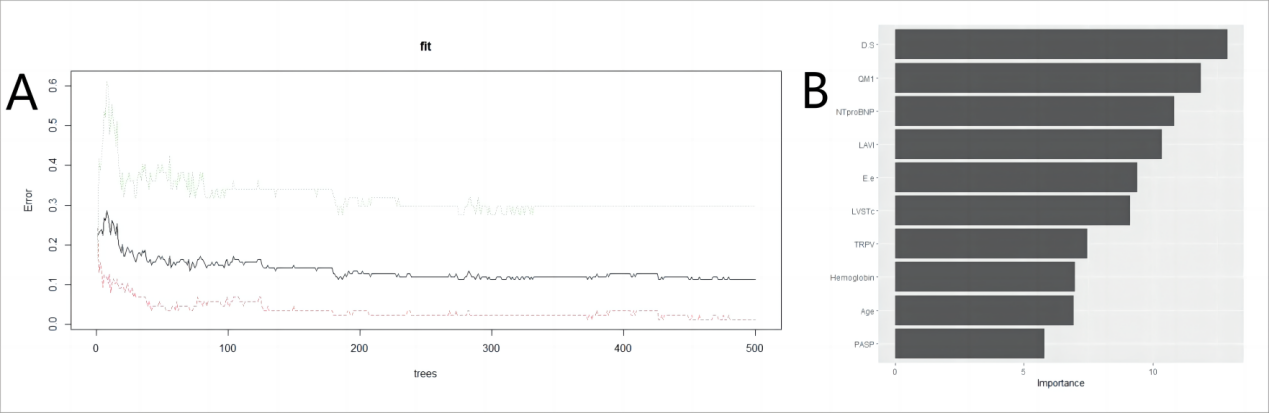


A is the relationship between tree number and OOB, B shows the partial variable importance of random forest in HFpEF.


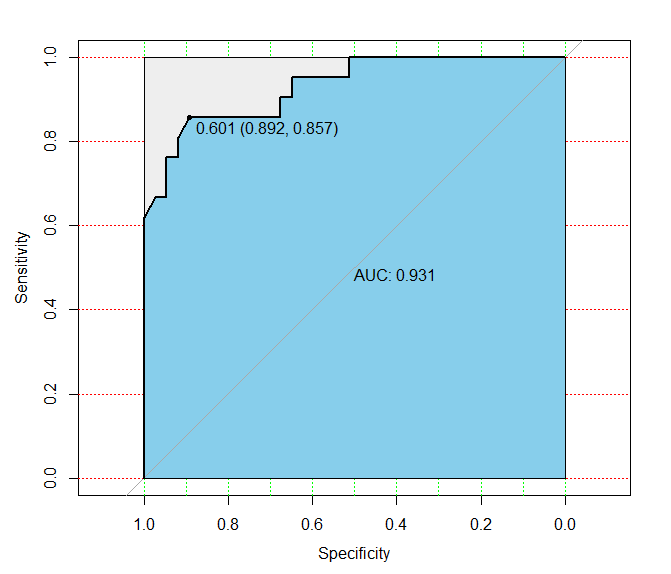
Random forest test set ROC curve. As a result, the nomogram model (AUC=0.945) was slightly more predictive than the random forest model (AUC=0.931), with an AUC increase of 0.014 (P>0.05).

**Supplementary References**

**[1] Reddy YNV, Carter RE, Obokata M, Redfield MM, Borlaug BA. A Simple, Evidence-Based Approach to Help Guide Diagnosis of Heart Failure With Preserved Ejection Fraction. Circulation 2018;138:861-870.**

**[2] Pieske B, Tschope C, de Boer RA et al. How to diagnose heart failure with preserved ejection fraction: the HFA-PEFF diagnostic algorithm: a consensus recommendation from the Heart Failure Association (HFA) of the European Society of Cardiology (ESC). Eur Heart J 2019;40:3297-3317.**

**[3] Nagueh SF, Smiseth OA, Appleton CP, Byrd BF 3rd, Dokainish H, Edvardsen T, Flachskampf FA, Gillebert TC, Klein AL, Lancellotti P, Marino P, Oh JK, Alexandru Popescu B, Waggoner AD, Houston T, Oslo N, Phoenix A, Nashville T, Hamilton OC, Uppsala S, Ghent Liege B, Cleveland O, Novara I, Rochester M, Bucharest R, St. Louis M. Recommendations for the evaluation of left ventricular diastolic function by echocardiography: an update from the American Society of Echocardiography and the European Association of Cardiovascular Imaging. Eur Heart J Cardiovasc Imaging 2016;17:1321–13**
